# Point-of-Care Testing for Hepatitis Viruses: A Growing Need

**DOI:** 10.3390/life13122271

**Published:** 2023-11-28

**Authors:** Matthew D. Pauly, Lilia Ganova-Raeva

**Affiliations:** Division of Viral Hepatitis, National Center for HIV, Hepatitis, STD, and TB Prevention, Centers for Disease Control and Prevention, 1600 Clifton Rd., NE, Atlanta, GA 30329, USA; mdpauly@emory.edu

**Keywords:** viral hepatitis, point of care, diagnosis, linkage to care

## Abstract

Viral hepatitis, caused by hepatitis A virus (HAV), hepatitis B virus (HBV), hepatitis C virus (HCV), hepatitis D virus (HDV), or hepatitis E virus (HEV), is a major global public health problem. These viruses cause millions of infections each year, and chronic infections with HBV, HCV, or HDV can lead to severe liver complications; however, they are underdiagnosed. Achieving the World Health Organization’s viral hepatitis elimination goals by 2030 will require access to simpler, faster, and less expensive diagnostics. The development and implementation of point-of-care (POC) testing methods that can be performed outside of a laboratory for the diagnosis of viral hepatitis infections is a promising approach to facilitate and expedite WHO’s elimination targets. While a few markers of viral hepatitis are already available in POC formats, tests for additional markers or using novel technologies need to be developed and validated for clinical use. Potential methods and uses for the POC testing of antibodies, antigens, and nucleic acids that relate to the diagnosis, monitoring, or surveillance of viral hepatitis infections are discussed here. Unmet needs and areas where additional research is needed are also described.

## 1. Introduction

Hepatitis, or liver inflammation, can be caused by lifestyle choices such as alcohol overconsumption or drug use, as well as genetics or viruses. Viral hepatitis is the most common and is the result of an infection with hepatitis A virus (HAV), hepatitis B virus (HBV), hepatitis C virus (HCV), hepatitis D virus (HDV), or hepatitis E virus (HEV), as well as other viruses. Each of the five hepatitis viruses belongs to a different virus family and is distinct in its transmission, replication, and interaction with its infected host. HAV and HEV are typically transmitted via the fecal–oral route and spread via contaminated food and water sources [1], but recent outbreaks demonstrated that HAV can also be transmitted parenterally [2]. HAV and HEV infections are generally self-limiting and resolve within 6 months after exposure [3,4]. HBV, HCV, and HDV are all blood-borne and transmitted via exposure to blood or other bodily fluids from infected persons [5]. Signs and symptoms during the acute phase of infection, which lasts for up to 6 months, are generally similar among the five hepatitis viruses and can include jaundice, abdominal pain, fever, aches, dark urine, and clay-colored stool. However, not all hepatitis virus infections cause noticeable signs or symptoms. Infections that persist for longer than six months are considered chronic. Chronic infections are typically only associated with HBV, HCV, and HDV and can lead to severe liver complications including cirrhosis, liver cancer, and liver failure [5,6,7].

The global burden of viral hepatitis is high. Together, the five hepatitis viruses cause tens of millions of infections each year [8,9,10,11]. More than one million global deaths are attributed to chronic HBV and HCV infections each year [9]. These infections and deaths occur despite the availability of vaccines for HAV and HBV and curative therapeutic drugs for HCV [12,13,14]. Many viral hepatitis infections are undiagnosed, and most published prevalence numbers are based on estimates. Significant proportions of infected individuals may be asymptomatic for prolonged periods and unaware of their infection. Without a diagnosis, infected persons cannot be linked to care and may continue to engage in behaviors that could transmit their infection to others. To alleviate the health burdens caused by viral hepatitis, the World Health Organization (WHO) set goals that include 90% reductions in the number of new HBV and HCV cases and 65% reductions in HBV- and HCV-associated deaths by the year 2030 [15]. Meeting these goals and decreasing the burden of HAV, HDV, and HEV will require improvements in prevention, diagnosis, and linkage to care. Point-of-care (POC) testing for various markers of hepatitis virus infections, immunity, or contamination is an attractive approach that could facilitate achieving these goals.

## 2. Point-of-Care Testing

### 2.1. Overview

Point-of-care testing can be performed at or near the patient or sample collection site. Typically, POC tests can be performed outside of a laboratory, with minimal or no equipment, and without extensive operator training [16]. Ideally, such testing yields results while the patient waits. Rapid diagnostic tests (RDTs) are a class of tests that produce results quickly, usually in 30 min or less and are often, but not always, compatible with POC use. While POC tests for certain infection markers have existed for decades, it was the COVID-19 pandemic that demonstrated the power of POC testing to improve infectious disease testing access and uptake on a large scale [17]. Most POC tests that have been developed for infectious diseases detect antibody or antigenic markers, but technologies for nucleic acid detection are improving rapidly.

To guide the development of tests that would meet the needs of health care systems in the developing world, the WHO’s Sexually Transmitted Disease Diagnostics Initiative released the ASSURED criteria in 2004, which encourage new diagnostic tests to be Affordable, Sensitive, Specific, User-friendly, Rapid and robust, Equipment-free, and Deliverable [18]. These criteria have been adopted to describe an ideal POC test. Updated REASSURED criteria that include Real-time connectivity and Ease of sample collection were proposed in 2019 [19]. The precise target criteria for what is considered an appropriate POC test will vary based upon the disease marker and the population being tested. Affordable POC tests should have per-sample costs below those of comparable laboratory tests. Tests with low costs will have greater opportunities for improving widespread access to care. When estimating affordability, the costs of any personnel, infrastructure, and necessary equipment needs to be accounted for. Sensitivity and specificity describe how well a test correctly classifies a sample as positive or negative for the tested analyte. These performance characteristics should be as close as possible to 100%; however, acceptable sensitivity and specificity will depend on the intended use of the test andthe clinical actions that the test result will be used to inform. Pretest and posttest probabilities should be taken into consideration when assessing whether a particular POC test is appropriate. The use of tests with low sensitivity or specificity may have different implications for the interpretation of results, depending on the prevalence of a particular infection within the population being tested. The number and duration of manual user steps, the duration and complexity of required user training, and the ease of interpreting results determine the user-friendliness of a test. POC tests that are easy to conduct and interpret maximize testing throughput and streamline results reporting. Rapid testing, which can be completed in less than 60 min, will promote the delivery of testing results while the tested individual waits and potentially allows for immediate clinical intervention wherever needed. Robust POC tests are unaffected by less-than-ideal conditions that could be experienced during transportation, storage, and operation of the test. The equipment required to perform a test should be minimized to reduce costs and increase access. Ideally, POC tests would not require any equipment, electricity, or other infrastructure. For a POC test to be effective in improving testing access, it needs to be deliverable to the end user without logistical, financial, or other burdens. In today’s world, most technology is connected via the internet. Real-time connectivity for POC tests could improve quality assurance by monitoring test runs and standardizing result interpretation as well as allowing for reporting test results to a provider or health care facility. POC testing will be most user- and patient-friendly when there is ease of sample collection. Where possible, sample materials that are less invasive to collect, like fingerstick blood or saliva, should be used in place of venous whole blood, serum, or plasma for POC tests. When judging the suitability of a POC test based upon these criteria, it is important to keep in mind the potential clinical impact of its use for a particular population or purpose. For instance, high sensitivity would be critical for POC tests that are intended to assess the attainment of a sustained viral response after therapy. On the other hand, high specificity, low cost, and ease of use may be a more important test criterion for surveillance or diagnosis in general population settings.

Traditional laboratory assays for the detection of antibody, antigen, and nucleic acid markers are well established and represent the standards against which novel techniques are judged. Serological assays for antibody or antigenic markers are commonly performed either manually or on automated testing platforms [20]. The standard for detection of nucleic acids is via quantitative polymerase chain reaction (qPCR) or quantitative reverse transcription polymerase chain reaction (qRT-PCR) [21]. These laboratory-based methods have high sensitivity and specificity, but they are often expensive and time-consuming and require extensive equipment and training. Therefore, POC tests that overcome some of these drawbacks are attractive as a way to increase access to diagnostic testing.

Point-of-care tests have been developed to detect various markers of viral hepatitis infections (Table 1). Point-of-care tests for the detection of HAV-specific antibodies or nucleic acids could assist in the rapid identification of food-borne outbreaks and help to inform mitigation efforts. Currently, the CDC recommends hepatitis B testing of all adults aged 18 years and older at least once in their lifetime using a multianalyte test that includes HBV surface antigen (HBsAg) and antibody markers. Point-of-care tests could potentially make this screening, as well as more frequent HBsAg testing among high-risk individuals, more accessible and allow for earlier diagnosis of an infection. The standard testing algorithm for HCV includes initially testing for HCV-specific antibodies, followed by reflex testing for HCV RNA, if positive. The use of POC tests for these markers of HCV infection would allow this testing to be accessible outside of the laboratory and for a faster diagnosis and linkage to care. The identification of HDV infections, which occur simultaneously with some HBV infections, could be an attractive use of RDTs due to their ability to be multiplexed for the detection of multiple antibodies and/or antigens. Like HAV, the rapid detection of HEV infections using POC tests could streamline outbreak identification and ensuing mitigation efforts. Here, we will describe the current testing approaches that are compatible with the detection of antibodies, antigens, and nucleic acids in a POC format and where future development and evaluation efforts should be directed. We will also highlight POC tests that have been approved by the U.S. Food and Drug Administration (FDA) or prequalified by the WHO and summarize their performance characteristics and impact in real-world testing situations (Table 2).

### 2.2. Lateral Flow Tests

**Figure 1:**
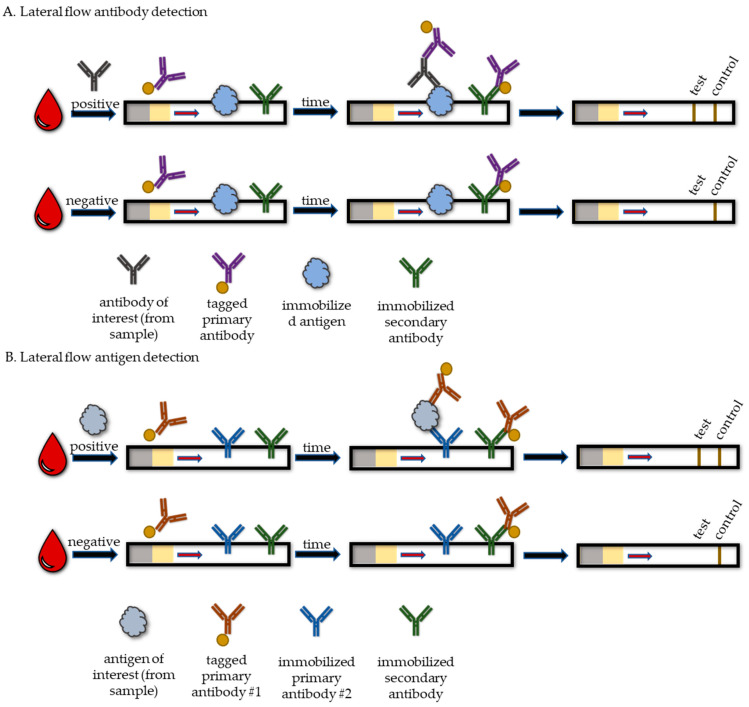
Lateral flow tests for detection of antibodies and antigens. (**A**) In antibody detection assays, sample is added to the sample pad (gray). Assay solution and sample flow across the conjugate pad (yellow), which contains primary antibodies tagged with a detection molecule (purple), to the reaction membrane containing immobilized antigen at the test line (light blue) and immobilized secondary antibody (green) at the control line. If the antibody of interest is present within the sample, it will be bound by the tagged primary antibody and bind to the immobilized antigen, producing a detectable band at the test line. (**B**) In tests for the detection of antigens, sample, which is added to the sample pad (gray), mixes with tagged primary antibody #1 (orange) on the conjugate pad (yellow). The sample and conjugate flow across the reaction membrane containing immobilized primary antibody #2 (blue) at the test line and immobilized secondary antibody (green) at the control line. Antigen (gray) present in the sample will become sandwiched between the tagged primary antibody and the immobilized primary antibody, allowing for a detectable band at the test line. For both antibody and antigen detection assays, a valid test will have a detectable band at the control line where the tagged primary antibody is bound by the immobilized secondary antibody.

Lateral flow tests (LFTs), which use immunochromatography, are the most common format for POC RDTs that detect the presence of antibodies or antigens [22]. These tests are similar to laboratory assays for antibody and antigen detection except that they are performed on lateral flow strips, do not involve liquid transfers or washes, and they produce results that can be interpreted visually without the need for an instrument (Figure 1). Lateral flow strips are composed of a sample pad, conjugate pad, reaction membrane, and an absorbent pad. The sample pad is where the sample is added, and the conjugate pad contains the detection molecule, conjugated to a tag. The tag is often a colored particle, such as colloidal gold or latex, but fluorescent and enzymatic tags can also be used [23,24]. The reaction membrane, usually made from nitrocellulose, allows the sample and detection molecule to flow via capillary action towards the absorbent pad and across the capture molecules that are immobilized at the test and control lines on the reaction membrane. If the tested analyte is present, a colored band will develop at the test line. If the test is operating properly, a colored band will be visible at the control line. Lateral flow tests for antigens usually take 10 to 30 min to produce a result and are hands-off after the sample is added.

The sample type and the detection and capture molecules used in LFTs differ based upon the analyte of interest. For LFTs that detect antibodies, samples are generally blood, serum, plasma, or saliva. The detection molecule that is conjugated to colored particles is typically a primary antibody that is specific for the immunoglobulin G (IgG) or immunoglobulin M (IgM) of the species that the sample came from, such as antihuman IgG if human samples are being tested. The detection antibody will bind to antibodies in the sample as it flows over the conjugate pad towards the reaction membrane (Figure 1A). The test line contains immobilized antigen that will be specifically bound by the antibodies that the test is designed to detect. For example, HCV antigens would be immobilized at the test line for the detection of anti-HCV antibodies. Antibodies specific for the antigen will bind to it and the tagged detection antibodies that are bound to the sample antibodies will be retained at the test line, creating an observable band. This process is analogous to an indirect immunoassay. The control line contains an immobilized molecule that will capture tagged detection antibodies that flow past the test line, allowing for an observable band to develop. For example, the control line may have an immobilized secondary antibody that is specific to the immunoglobulin of the species that the detection antibody is from. Variations of this approach are common, but they follow the same general principles for capturing the desired antibodies from the sample.

Lateral flow tests that detect antigens include a conjugate pad containing primary antibodies that are specific for the antigen of interest and are conjugated to colored particles (Figure 1B). Antibodies that are specific for the antigen of interest will also be immobilized at the test line. Specific antigens present in the sample will be sandwiched between these primary antibodies and result in a colored band developing at the test line. As in LFTs that detect antibodies, the control line contains antibodies that will specifically bind the primary antibody from the conjugate pad. If the antigen from the sample is inaccessible due to a higher-order molecular structure or the antigen existsin antibody–antigen complexes, it may be necessary to process samples with heat or chemicals before testing to ensure accurate detection.

### 2.3. POC Nucleic Acid Tests

**Figure 2:**
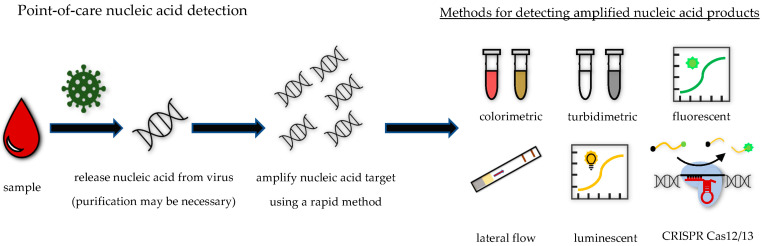
Detection of nucleic acids at the point of care. Simple-to-collect sample types, such as fingerstick blood, are used for POC testing. Depending on the test being used, pre-extraction processing, such as plasma separation from whole blood, may be required. To release nucleic acids from virus in the sample, simple processes such as heating, osmotic stress, or a chemical lysis buffer can be used. Concentration of extracted nucleic acids or removal of amplification inhibitors present within the sample can improve assay performance. These processes are achieved using purification methods such as magnetic bead- or silica membrane-based techniques. Approaches for the rapid amplification of nucleic acids that are attractive for point-of-care use include isothermal methods such as LAMP and RPA and rapid RT-PCR techniques. Amplified nucleic acids can be detected via a variety of techniques that can be measured visually or with simple equipment. These techniques include colorimetric-, turbidimetric-, fluorescent-, lateral flow-, luminescent-, and CRISPR-Cas12/13-based approaches.

Detection of nucleic acids requires three steps. First, nucleic acids need to be released from the viruses or cells harboring them. The extraction of nucleic acids can also include purification. Second, the extracted target nucleic acids need to be amplified to increase the amount present within the sample. Third, the amplified nucleic acids of interest need to be detected. Streamlining these processes for a POC format, while maintaining appropriate sensitivity and specificity characteristics, is challenging (Figure 2).

Methods for extracting nucleic acids include heat, osmotic stress, and chemical lysis [25,26,27,28,29]. These approaches are generally compatible with a POC format but may require equipment such as heating devices or considerations for the use and disposal of hazardous chemicals found in lysis buffers. Body fluid samples contain molecules that can inhibit or interfere with downstream amplification and detection processes. Purification of the extracted nucleic acid from these inhibitory molecules are routine in the laboratory and can be performed using selective precipitation of nucleic acids or collecting them on solid-phase substrates such as silica membranes or magnetic particles [30]. However, adapting these approaches for use in POC formats is difficult due to the number of manual steps, length of time, and the requirement for equipment such as centrifuges. Magnetic microspheres with properties that allow for the capture and separation of nucleic acid sample materials may represent the most promising approach to sensitive POC applications [27,31]. Indeed, several simple devices for the automated processing of small-volume samples to purify nucleic acids based on magnetic bead separation technologies or other technologies have been reported [32,33,34]. Techniques for POC separation of plasma from whole blood prior to nucleic acid extraction that may allow for improved detection of nucleic acid targets from blood samples have also been developed [35,36,37,38].

The nucleic acid amplification methods used in POC tests need to be rapid. Isothermal methods are attractive because they produce large amounts of product in 10–30 min at a single temperature. The two most common isothermal methods are loop-mediated isothermal amplification (LAMP) and recombinase polymerase amplification (RPA). Both methods can be paired with a reverse transcriptase (RT) for the detection of RNA targets. LAMP reactions use four or six primers that are complementary to six or eight regions of the target nucleic acid [39,40]. In these reactions, polymerases with high strand displacement properties synthesize large DNA products containing hairpin loops when the temperature is maintained between 60 °C and 65 °C. In RPA reactions, recombinase and single-strand binding proteins allow for the rapid amplification of DNA at a temperature between 37 °C and 42 °C using two primers [41]. In addition to isothermal methods, rapid PCR techniques have also been developed, but these require the use of more complex devices [42,43,44,45].

There is a range of ways to detect amplified nucleic acid products that could be compatible with POC use. Equipment-free methods include colorimetric, lateral flow, and turbidimetric detection. Colorimetric methods rely on a color change that occurs because of the presence of DNA amplification products, the production of pyrophosphate as a by-product of amplification, or the acidification of the reaction solution that occurs during amplification [46,47,48,49]. Colorimetric methods are non-specific and will detect any nucleic acid amplification, which can lead to specificity issues if the reaction amplifies non-target nucleic acids. Additionally, subtle color changes can be difficult to distinguish by eye [50]. Lateral flow strips can be used to detect amplified nucleic acids if the primers used for amplification contain tags such as biotin and fluorescein [41,51]. DNA products containing the tagged primers will result in the development of a positive test line band when they are captured by immobilized streptavidin at the test line and bound by fluorescein-specific antibodies that are conjugated to colored particles. Nucleic acid amplification reactions can yield large amounts of DNA product that will cause the solution to become turbid [52]. Turbidity can be judged by eye or measured with a turbidimeter.

Other methods for the detection of amplified nucleic acids, such as the measurement of fluorescence and luminescence, require equipment. Fluorescent methods require a fluorometer, and measurements can be obtained in real time or at the end of the amplification reaction. The generation of fluorescence can be specific or non-specific for the nucleic acid target. Non-specific fluorescent methods use molecules that are fluorescent only when double-stranded DNA is present [53]. Specific methods use nucleic acids tagged with fluorophores and quenchers that are specific for the nucleic acid target and fluoresce only after the successful amplification of that target [25,41,54,55]. Methods such as bioluminescent assay in real time (BART) require a luminometer to measure luminescence produced by luciferase [56]. In this approach, pyrophosphate produced as a by-product of nucleic acid amplification is converted into Adenosine Triphosphate (ATP) and used as a substrate by luciferase.

The adaptation of methods for the specific detection of target gene amplification using clustered regularly interspersed short palindromic repeat (CRISPR)-associated (Cas) proteins in POC formats has recently gained attention. These proteins originally evolved as a part of bacterial and archaeal adaptive immune systems for the purpose of destroying foreign nucleic acids. Nucleic acids that are complementary to trans-activating CRISPR RNA (crRNA) are specifically targeted by Cas proteins possessing nuclease activities. Two of these proteins, Cas12 and Cas13, have potential application for nucleic acid detection due to their non-discriminant DNase and RNase activity after recognizing their target nucleic acid. These applications include the DNA endonuclease-targeted CRISPR trans reporter (DETECTR) assay that uses Cas12 and the specific high-sensitivity enzymatic reporter unlocking (SHERLOCK) assay that uses Cas13 for the detection of DNA and RNA targets, respectively [57,58]. Both methods could allow for highly specific detection of amplified nucleic acids in a POC format when coupled with fluorescent or lateral flow read-outs.

### 2.4. Multiplex Tests

Multiplex tests, which can detect more than one test analyte simultaneously, may be advantageous in a POC setting, because they allow for the determination of more information about a patient’s health or a sample without additional time or effort. The signs and symptoms of infection with hepatitis viruses are difficult to distinguish in the absence of specific diagnostic tests for each of the five hepatitis viruses. The sample types used for these diagnostic tests include whole blood, serum, or plasma which can be used to test for antibodies, antigens, and nucleic acids. Non-hepatitis virus markers with similar risk characteristics and that use the same sample type, such as HIV or antibody markers from blood and enteric viruses from stool, may also be included in multiplex tests to increase the breadth of what can be evaluated in a single test. Some multiplex POC tests involving hepatitis virus markers are mentioned in the sections below. A drawback of multiplex tests is that there can be non-uniform performance for the detection of all analyte components.

## 3. Tests by Viral Agent

### 3.1. Hepatitis A Virus

Hepatitis A virus is a positive-sense RNA virus in the Picornaviridae family. It encodes 11 genes that are expressed as polyproteins [59]. The structural proteins form the virus capsid that encloses the genomic RNA in virions. Virions that are shed in the feces are non-enveloped, while virions that are shed in the blood can contain a lipid membrane that surrounds the capsid in a quasi-envelope [60]. Virions are stable and can remain infectious for months [61]. There are three genotypes of HAV (I, II, and IIII) that infect humans; however, there is only a single serotype.

HAV caused an estimated 159 million acute infections and nearly 4000 deaths globally in 2019 [8]. Children and adolescents generally exhibit few or no symptoms and resolve their infection without complications. Older adults, however, can experience severe disease. In countries with high endemicity, most people are infected early in life, develop an antibody response, and are protected from reinfection. In high-income countries, improved sanitation and hygiene have limited exposure to HAV, resulting in adult populations who are susceptible to HAV [62]. The primary route of HAV infection is via the ingestion of contaminated food or water and close contact with infected persons. Recently, unsafe injection drug and sexual practices have been recognized as additional modes of transmission in populations without preexisting immunity [63]. After infection, it typically takes between three and five weeks before any symptoms develop [3]. HAV is found in the blood and shed in the stool during infection. Antibodies appear within a few weeks after infection. IgM anti-HAV antibodies appear first and wane after the resolution of infection [3]. The presence of these IgM antibodies indicates a current or recently resolved HAV infection. IgG anti-HAV antibodies appear later during infection and rise to high titers that can persist for decades. An effective vaccine that protects against HAV infection exists and is recommended for children and at-risk adults [12]. Vaccination can also be used as a postexposure prophylactic if given within two weeks after exposure to HAV [64].

Diagnosis of HAV infection generally occurs after the presentation of signs of liver inflammation such as jaundice and elevated alanine aminotransferase (ALT) levels. HAV infections can be diagnosed via laboratory tests that detect the presence of IgM anti-HAV antibodies or HAV RNA. Immunity to HAV from infection or vaccination can be determined by the presence of IgG anti-HAV in the absence of IgM anti-HAV. Diagnosis of HAV infection at the POC or with a POC-compatible test could improve patient care and speed outbreak responses, promote interventions such as vaccination to decrease transmission, and allow for postexposure prophylactic vaccination of people who may have been exposed to HAV. However, there are currently no POC tests for markers of HAV infection or immunity that have been approved by the FDA or prequalified by the WHO.

Several rapid LFTs for the detection of IgG and/or IgM anti-HAV antibodies have been developed. An LFT that detects IgM anti-HAV from serum or plasma samples in 20 min was reported to have 100% sensitivity and 99% specificity when compared with an enzyme immunoassay for testing 150 samples from patients with acute HAV infections and 75 healthy individuals [65]. The Bioline HAV IgG/IgM Rapid Test (Abbott Diagnostics, Abbott Park, IL, USA), which allows for multiplex detection of both IgG and IgM anti-HAV from serum or plasma in 20 min, was evaluated against laboratory immunoassays using patient cohorts from Brazil, Burkina Faso, and India [66,67,68]. These reports suggest different performance characteristics for the detection of the two antibody isotypes. For IgG anti-HAV, the sensitivity was poor (49–67%) and the specificity was high (98–100%), while for IgM anti-HAV, the sensitivity was high (86–100%) but the specificity was lower (80–99%). Interestingly, when used in an outbreak setting, the specificity of IgG anti-HAV decreased to 21% [68]. This could indicate performance differences between the rapid test and the laboratory immunoassay used as a comparison for detecting early IgG seroconversion. Additionally, problems with interpreting results may be an issue with this test, as there was only 60–80% agreement among three observers [68]. The EuDx-HE (A,B,C) (Eudipia Inc., Cheongju, Republic of Korea) is a multiplex test that detects IgM anti-HAV, hepatitis B virus surface antigen (HBsAg), and anti-HCV from plasma in 15 min. Its performance in terms of detection of IgM anti-HAV is similar to the Bioline HAV IgG/IgM Rapid Test, with a sensitivity of 94.7% (95% CI, 85.4–98.9%) and a specificity of 99.4% (95% CI, 98.9–99.7%) [69]. As an alternative to using antigens for the detection of antibodies, proteinticles, which are nanoscale protein particles that display antigenic peptides on their surface, have been used to develop an LFT that is capable of multiplex detection of antibodies, targeting HAV, HCV, and HIV in 30 min [70]. The sensitivity for detection of total anti-HAV (IgG and IgM) using this assay was determined to be 100%, with a specificity of 100%, although this analysis included a relatively small number of samples. Most of the evaluations of LFTs for the detection of anti-HAV have been performed in laboratory setting using serum or plasma. Before these tests can recognize their potential for POC use to diagnose HAV infection by the presence of IgM anti-HAV or indicate immunity by the presence of IgG anti-HAV, their performance outside of the laboratory and with whole blood collected through fingerstick need to be evaluated.

Commercial tests for the POC detection of HAV RNA are not available; however, some laboratory-developed tests with characteristics that are attractive for use in settings with low resources have been developed. An RT-LAMP assay that has a similar limit of detection to qRT-PCR and can detect HAV genotypes IA, IB, and IIIA in 30–50 min using real-time fluorescence was reported [71]. However, this assay uses laboratory approaches for extracting and purifying HAV RNA from stool samples. An RPA assay that is capable of detecting HAV RNA from 0.5 mL of whole blood treated with a rapid nucleic acid extraction reagent in 30 min using lateral flow strips for visual interpretation of results was reported to have perfect sensitivity and specificity when evaluated using a small number of samples [72]. These examples suggest that a POC test for the detection of HAV RNA may be possible; however, additional developments and evaluations, especially related to using sample types and sample processing methods that are relevant to a POC format are needed.

Testing for markers of immunity or infection are not the only possible uses of POC tests related to HAV infection. The primary route of transmission for HAV is contaminated food and water. Testing food and water for HAV is uncommon, and when it does occur, it is often reactive and in response to an outbreak. Due to the length of time between HAV exposure and the onset of symptoms, the contaminated food or drink item is not usually available for testing. Simplifying food and water testing with POC-compatible formats would move testing outside of the laboratory and potentially allow for routine screening of items such as shellfish, leafy produce, and frozen vegetables that are commonly associated with outbreaks. An assay that uses RT-LAMP in combination with real-time bioluminescent detection was developed and shown to be capable of detecting low-level HAV contamination of green onions, strawberries, mussels, and milk [73]. However, the laboratory methods used for the processing of the samples and the length of time needed to perform the assay (>100 min) were not necessarily attractive for POC use. Additionally, since HAV contamination levels on food or in water are typically low, the limit of detection of POC HAV RNA tests may need to be improved to allow for testing that is sensitive enough to accurately identify foodstuffs that are contaminated with HAV.

### 3.2. Hepatitis B Virus

HBV is an enveloped virus with a 3.2 kilobase partially double-stranded DNA genome that is a member of the Hepadnaviridae family. HBV is divided into at least nine genotypes (A–I) [74]. It replicates via reverse transcription of a pregenomic RNA intermediate to produce its DNA genome [75]. Its virion is composed of the DNA genome within a capsid composed of the HBV core antigen (HBcAg) and enveloped in a lipid membrane that is studded with HBV surface antigen (HBsAg) [76]. Additionally, HBV e antigen (HBeAg) can be found in the blood of most infected people before partial immune control of viral replication occurs [77].

HBV is transmitted via percutaneous and perinatal routes of infection. An estimated 296 million people are currently infected with HBV, but only 10% of them have been diagnosed [78]. Major risk factors include being born to an HBV-infected mother, unsafe sexual behaviors, and exposure to contaminated blood or bodily fluids. Acute HBV infections are generally asymptomatic. When symptoms do occur, they are typically observed approximately 2 months after exposure to the virus. HBV DNA and HBsAg are observed in the blood within a few weeks of exposure and can be used to diagnose current HBV infection. Antibodies targeting HBcAg (total anti-HBc) first appear a few months after exposure. IgM anti-HBc are present during acute HBV infection but wane rapidly [79]. IgG anti-HBc, on the other hand, persists after the resolution of infection or during chronic infection. While total anti-HBc is a marker of exposure to HBV, these antibodies do not provide protection against HBV. Antibodies targeting HBsAg (anti-HBs) do provide protection against HBV infection and are present after vaccination or resolution of an HBV infection [13]. The probability of an HBV infection resolving during the acute phase varies with the age of the infected person. Most HBV infections in newborns (up to 95%) will become chronic, while less than 10% of HBV infections in adults will become chronic [80,81].

Chronic HBV infections can be categorized into stages based upon virus replication, HBeAg expression, and serum ALT levels, which are a marker of liver inflammation [80,81,82]. These stages can vary in length and progression. The immune-tolerant (HBeAg-positive chronic infection) stage is categorized by high HBV DNA levels, HBeAg expression, and normal ALT levels. During the immune-active (HBeAg-positive chronic hepatitis B) stage, high HBV DNA levels and HBeAg expression remain, but the targeting of the HBV infection by the immune system leads to liver inflammation and elevated serum ALT levels. The inactive carrier stage (HBeAg-negative chronic infection) occurs when antibodies targeting HBeAg (anti-HBe) appear and promote control of HBV replication. During this stage, HBV DNA and ALT levels are low, HBeAg is undetectable, and the probability of transmission is low, even during childbirth [82]. Anti-HBe can select for HBV mutants that downregulate or prevent HBeAg expression and can promote increased HBV replication and liver inflammation during the HBeAg-negative chronic hepatitis B stage [83]. Achieving undetectable HBsAg expression during the HBsAg-negative stage, with or without anti-HBs, is a good prognostic indicator for low risk of new HBV-associated liver damage [84].

The best preventative measures for HBV infections are vaccination and avoiding risky behaviors. HBV vaccination shortly after birth can reduce the probability of mother-to-child transmission [85]. There is no cure for chronic HBV infections, and available therapeutics often only suppress viral replication without leading to HBsAg seroconversion. These therapeutics for HBV infection include pegylated interferon alpha and nucleos(t)ide analog drugs (NUCs), such as entecavir and tenofovir [78,81,83]. Due to the risk of reactivation of HBV replication after ending therapy, NUCs often need to be taken for extended periods of time [86,87].

Many POC tests for the detection of HBsAg are commercially available and routinely used in certain settings. Rapid HBsAg tests are typically compatible with whole blood, plasma, or serum samples and provide a result in less than 20 min. These types of tests generally exhibit good performance when compared with laboratory tests. Indeed, a meta-analysis that included 30 studies evaluating the performance of 33 different HBsAg LFTs found an overall sensitivity of 90.0% (95% CI, 89.1–90.8%) and specificity of 99.5% (95% CI, 99.4–99.5%) [88]. More recent evaluations of commercial HBsAg LFTs have shown sensitivities above 91% and specificities above 94% [89,90,91]. Mutations within the antigenic determinants of HBsAg appear to have little impact on the performance of certain HBsAg LFTs, although this may depend on the specific antigen epitopes that are recognized by the antibodies used in the test [92]. Lateral flow tests have also been developed that are capable of differentiating HBV genotypes A, B, C, and D using genotype-specific antibodies that are immobilized at multiple test lines [23]. The performance of HBsAg detection remains high when multiplexed with the detection of antibodies targeting HAV, HCV, or HIV [69,93,94]. Point-of-care HBsAg testing has been demonstrated to reduce testing costs and improve linkage to care compared with traditional laboratory testing for certain populations with high burdens of HBV infection [95].

Rapid tests for the detection of HBeAg are available, but they generally have poor sensitivities ranging from 29.8% to 82%, making them impractical for accurately assessing the stage of chronic HBV infection or evaluating the risk of mother-to-child transmission at the POC [96,97,98,99]. However, a recently reported LFT using nanoparticle complexes to amplify the test-line signal exhibited high sensitivity and specificity for the detection of HBeAg [100]. Rapid tests for antibody markers that are related to HBV infection have also met with limited success. POC tests for anti-HBs could be used to assess HBV vaccine uptake, immunity, or resolution of HBV infections, but the LFTs that have been reported for this analyte have poor sensitives that range from 50.4% to 69.5% and specificities between 93.0% and 98.4% [101,102,103,104]. Similarly, LFTs for the detection of anti-HBeAg could be used to identify patients in a stage of partial immune control of their HBV infections at the POC, but reported tests have sensitivities in the 45.2% to 82.8% range [97,102,103].

Although HBsAg LFTs work well for the POC diagnosis of current HBV infection, POC tests for HBV DNA could be useful for monitoring antiviral therapy and informing decision making regarding HBV therapy. Several approaches have been used to develop HBV DNA tests that are rapid, inexpensive, and could be compatible with POC use. Several LAMP tests that are specific for HBV DNA have been developed. A recent meta-analysis of nine such tests that can be completed in under 60 min showed that they had a pooled sensitivity of 91% (95% CI, 89–92%) and specificity of 97% (95% CI, 94–99%) compared with conventional PCR-based tests [105]. Recombinase polymerase amplification assays have also shown promise for the rapid detection of HBV DNA. These RPA-based tests are typically faster than LAMP-based tests and have limits of detection of 10–1000 HBV DNA copies per reaction when used with real-time fluorescence or lateral flow strip detection methods [106,107,108]. These methods are compatible with the multiplex detection of a control DNA that is spiked into the sample to verify that the RPA reaction is working [109,110]. Combining RPA and LAMP in a method known as Penn-RAMP may improve the sensitivity and speed of HBV DNA detection compared with either method alone [111]. Additional isothermal methods have also been reported to work well for amplification of HBV DNA [112,113]. Both LAMP and RPA have been successfully coupled with Cas12- or Cas13-based methods to improve the sensitivity and specificity of HBV DNA detection via real-time fluorescence or lateral flow strips within 60 min [114,115]. A LAMP- and Cas12-based detection method was able to discriminate HBV genotypes B and C and may be adapted to identifying other genotypes [116].

Evaluations of rapid methods for HBV DNA detection often use commercial nucleic acid extraction kits with plasma or serum samples, which are poorly compatible with point-of-care sample collection and use. However, a few studies using plasma or whole blood samples with simple heating or lysis buffer methods for nucleic acid extraction have found decreased sensitivity of HBV DNA detection in LAMP or RPA reactions [106,117,118]. One such study evaluating simple boil-and-spin or bead-based extraction methods with serum samples found that these approaches have high sensitivity and specificity to diagnose HBV DNA levels above 200,000 IU/mL, which is a relevant clinical threshold to treatment recommendations for preventing mother-to-child HBV transmission [119].

The Xpert HBV Viral Load Test on the GeneXpert instrument (Cepheid, Sunnyvale, CA, USA) is the only commercially available rapid test for HBV DNA that has been publicly evaluated. The test provides quantitative HBV DNA levels in under 60 min with minimal user intervention but is only marketed for use with serum or plasma samples. It has a limit of quantitation of 10 IU/mL, and its results correlate well with automated laboratory qPCR tests [120,121]. With this test, dried blood spots have been shown to be a suitable sample type that provide results that correlate well with plasma samples [122,123].

### 3.3. Hepatits C Virus

HCV is a small, enveloped virus from the Flaviviridae family that has a 9.6 kb positive-sense RNA genome. HCV is classified into at least eight distinct genotypes (1–8), with multiple subtypes in each [124]. The genetic material is expressed as a single polyprotein that is post-translationally processed into 10 structural and non-structural (NS) proteins [125]. Virions are composed of the RNA genome, complexed with a capsid made of core antigen (cAg) and enclosed in a lipid membrane studded with envelope proteins 1 and 2 (E1 and E2) [126].

HCV infects an estimated 58 million people worldwide, with around 1.5 million new infections each year, but only 21% of them have received a diagnosis [9]. HCV is transmitted percutaneously by contaminated blood and bodily fluids. Major risk factors for HCV infection include having received a transfusion prior to routine screening of the blood supply in the early 1990s and participating in unsafe injection practices [127]. After exposure to the virus, HCV RNA and cAg can be detected in the blood in one to two weeks. Fewer than 30% of HCV-infected people exhibit symptoms, which can appear by six weeks. Antibodies targeting HCV proteins (anti-HCV) can be first observed between 4 and 12 weeks after exposure but do not provide protection from current or future HCV infections [128]. Antibody responses vary among infected persons, but most commonly target cAg, NS3, NS4a, NS4b, and NS5a. More than half of HCV infections will persist for longer than six months and become chronic. Chronic infections can last for decades and are associated with increased risk of liver cirrhosis and cancer [6]. Fortunately, antiviral drugs that target the NS3/NS4a protease, NS5a, or the NS5b polymerase and have >95% cure rates after 12–24 weeks of treatment are available [14,129]. No vaccines that can prevent HCV infection have been developed, and the best way to prevent infection is to avoid risky behaviors.

**Figure 3:**
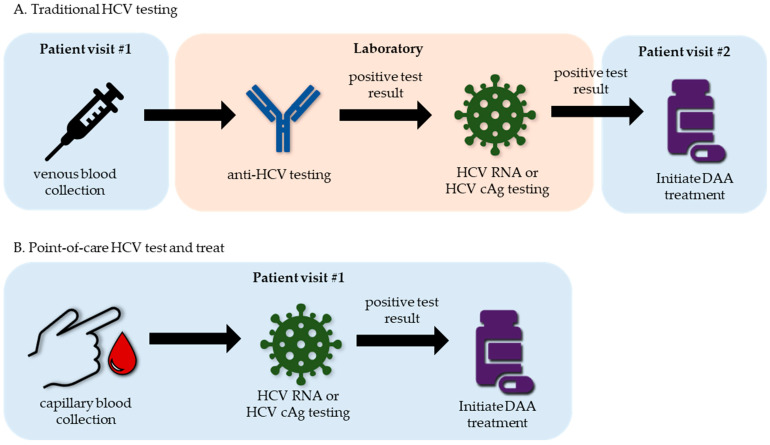
Point-of-care tests could simplify HCV diagnosis and linkage to care. (**A**). Traditional algorithms for the diagnosis of HCV infections require venous blood draw specimens to be sent to a laboratory for anti-HCV testing. Samples positive for anti-HCV are reflex-tested for HCV RNA to diagnose current HCV infections. Patients need to return to their health care provider to receive a positive HCV infection diagnosis and to be linked to treatment. This requirement for an additional visit to a health care provider can result in patients never receiving a diagnosis or being linked to treatment. Additionally, some people may have poor access to testing due to geography or cost. (**B**). Point-of-care tests for HCV RNA or cAg from capillary blood could alleviate some of the issues with current testing algorithms by making HCV diagnostics less expensive, able to be performed in more locations, and rapid. Tests with these features may make test-and-treat diagnostic models possible, allowing for testing, diagnosis, and treatment initiation to occur in a single visit to a health care provider.

Traditional diagnostic algorithms test for anti-HCV followed by reflex testing anti-HCV-positive samples for HCV RNA or cAg to identify current HCV infections [130]. This approach economizes testing but will miss infections in people who have not yet developed anti-HCV antibodies. Since testing for current HCV infections is performed in a laboratory, access to testing can be an issue for some populations, and the need for health care provider follow-up, which is required to receive a diagnosis, could lead to patients never being linked to care [131]. Using rapid and inexpensive POC tests to diagnose HCV infections while a patient waits could allow for test-and-treat diagnostic algorithms that reduce access barriers and promote linkage of infected persons to care [132] (Figure 3). Since HCV RNA and cAg are the only markers for diagnosing a current HCV infection, reliable POC tests that detect them will be needed to allow for test-and-treat diagnostic strategies. The WHO recommends 3000 IU/mL as an acceptable target for POC HCV RNA tests [133].

A few commercially available tests that can rapidly extract and detect HCV RNA via quantitative RT-PCR have been developed. Thorough evaluations have been reported for the Genedrive HCV ID Kit (Genedrive, Manchester, UK), Xpert HCV Viral Load kit (Cepheid, Sunnyvale, CA, USA), and Xpert HCV VL Fingerstick kit (Cepheid, Sunnyvale, CA, USA), which have received WHO prequalification, but not FDA approval. The Genedrive HCV ID Kit can detect HCV RNA from 30 µL of plasma or serum in 90 min. This test can be operated outside of a laboratory but involves 12 manual steps. Its limit of detection is 2362 IU/mL, it can detect the most common HCV genotypes and subtypes, and its sensitivity and specificity compared with laboratory HCV RNA detection methods range from 96.2 to 100% and 99.5 to 100%, respectively [134,135,136]. Similarly, the Xpert HCV Viral Load kit that operates on the Cepheid GeneXpert instrument detects HCV RNA from plasma samples in 105 min but requires no manual steps after addition of the sample to the test cartridge. This test has a lower limit of quantitation of 10 IU/mL and good quantitative correlation, sensitivity, and specificity compared with laboratory HCV RNA detection methods [137,138,139]. While neither of these tests are truly POC in nature due to the need for plasma or serum samples and their run times are greater than one hour, their speed, simplicity, and equipment requirements make them attractive alternatives to traditional laboratory methods. The existing commercial test with the best applicability for POC HCV diagnosis is the Xpert HCV VL Fingerstick kit that is also run on the GeneXpert instrument. This test detects HCV RNA as low as 40 IU/mL and quantifies HCV RNA as low as 100 IU/mL from 100 µL of fingerstick blood in 60 min [140]. A recent meta-analysis including seven published evaluations of this test found a pooled sensitivity of 99% (95% CI, 97–99%) and specificity of 99% (94–100%) compared with paired plasma or serum samples tested using laboratory HCV RNA tests [141]. Eighty percent of positive results from this test are obtained within 40 min, potentially allowing for an even faster diagnosis of HCV infections [142]. However, potential drawbacks of the Xpert HCV VL Fingerstick kit are the occasional return of invalid test results and costs that may be prohibitive in certain markets [141,143].

Several RT-LAMP assays for the detection of HCV RNA that have limits of detection and sensitivities similar to qRT-PCR assay with amplification times under 60 min have been reported [144,145,146,147,148,149]. The performance of these methods has typically only been reported using nucleic acids that are purified from serum or plasma samples using commercial nucleic acid extraction kits that are poorly compatible with POC use due to time and equipment needs. In studies that did evaluate simpler nucleic acid extraction methods, heat treatment of plasma samples exhibited poor sensitivity, while magnetic-bead-based purification approaches looked promising [150,151]. Several devices to simplify the amplification and detection of HCV RNA using RT-LAMP have been developed. These include portable battery-operated LED heaters integrated with ion sensors to detect nucleic acid amplification and heat-block-compatible microfluidics devices that integrate amplification and lateral flow detection of HCV RNA as low as 398 copies per reaction in under 40 min [152,153]. A microfluidics device that is capable of integrating magnetic-bead-based nucleic acids extraction from plasma samples with RT-LAMP and colorimetric detection was reported to be able to detect 500 HCV virions per mL in 45 min [33]. Microfluidics devices have also been developed for the multiplex LAMP detection of HBV, HCV, and HIV nucleic acids [154]. Fluorescent and lateral flow detection of HCV RT-LAMP products via a Cas12-based method was reported to have 96% sensitivity and 100% specificity compared with a reference RT-PCR test with a runtime of 60–90 min [155].

Recombinase polymerase amplification has also been applied to the detection of HCV RNA. A pan-genotypic RPA test was reported to have a limit of detection of 25 copies per reaction with 100% sensitivity and specificity compared with qRT-PCR, although separate reverse transcription and RPA reactions were needed to achieve this performance [156]. A one-step RT-RPA that integrates the two reactions was reported to detect 10 copies of HCV RNA per µL in 30 min using a lateral flow strip, but the genetic region targeted by the RPA primers is unlikely to broadly detect the range of HCV genotypes [157]. Combining RT-RPA with LAMP may allow for faster and more sensitive detection of HCV RNA than RT-LAMP alone [108]. Other isothermal techniques, including catalytic hairpin assembly and polymerase spiral reaction, have been demonstrated to work for sensitive and rapid visual detection of HCV RNA [158,159]. However, before any isothermal nuclei acid amplification techniques can be considered POC tests for HCV RNA, simple methods for extracting HCV RNA from fingerstick whole blood samples will need to be developed.

Detection of HCV cAg indicates current HCV infection, but commercial POC tests for this marker have not been developed. A few LFTs have been reported for the detection of HCV cAg from serum or plasma in 15–20 min [160,161]. However, further development for using these types of tests with fingerstick blood samples and samples from people who have antibodies targeting HCV cAg will be necessary to support their use at the POC.

Detection of anti-HCV is the first step in traditional HCV diagnostic algorithms. However, anti-HCV cannot be used to diagnose current HCV infections and is used only to screen for which patients or samples should have follow-up testing for HCV RNA or cAg. Testing for anti-HCV at the POC would not be compatible with test-and-treat models but could simplify screening samples to determine which ones require HCV RNA reflex testing using a laboratory or POC test. Anti-HCV LFTs are widely available and several tests have WHO prequalification, including the OraQuick HCV test (Orasure, Bethlehem, PA, USA), which is FDA-approved. Two meta-analyses evaluating the published use of anti-HCV LFTs with whole blood, serum, or plasma samples found pooled sensitivities of 97.4% (95% CI, 95.9–98.4%) and 98% (95% CI, 98–100%) and specificities of 99.5% (95% CI, 99.2–99.7%) and 100% (95% CI, 100–100%) [162,163]. Similarly, a recent large-scale evaluation of seven anti-HCV LFTs with serum or plasma samples found sensitivities ranging from 97.2 to 100% and specificities above 99.5% for all but one test [164]. Some anti-HCV LFTs, including the OraQuick HCV Test, are approved for use with saliva or oral fluid samples. The sensitivity for the detection of anti-HCV from saliva or oral fluids is poorer than from blood, serum, or plasma, but may still be adequate for screening in high-prevalence settings such as drug clinics or needle exchange centers [160,162,165]. Anti-HCV LFTs can be multiplexed with other markers of viral infection. Several multiplex tests have been developed that can detect anti-HAV, HBsAg, and anti-HIV while maintaining sensitive and specific detection of anti-HCV [69,70,93,94].

Point-of-care testing used in the diagnosis of HCV infections could allow for health care cost savings and improved patient perception of the testing process. In high-prevalence settings, such as among intravenous drug users, POC HCV RNA tests may represent a cost-saving alternative to traditional laboratory diagnostic algorithms that also allows for earlier diagnosis of HCV infection [166,167]. However, in most settings with an anti-HCV prevalence below 74%, POC anti-HCV testing followed by reflex testing using POC HCV RNA testing may have the lowest cost per diagnosis and treatment initiation [168]. Similarly, in low- and middle-income countries (LMICs), POC anti-HCV testing followed by POC HCV RNA may be the most cost-effective strategy, unless POC HCV RNA test costs decline [169]. Even with the cost savings associated with POC testing, current test pricing may make elimination goals difficult to afford for many LMICss. An additional advantage of these testing strategies is that POC anti-HCV and HCV RNA testing using fingerstick blood samples is generally well regarded by patients in terms of ease, acceptability, and preference compared with venipuncture [170,171]. A large evaluation of the cost-effectiveness of HCV self-testing in four different countries demonstrated that self-testing was not cost-efficient, but that it significantly increased the number of diagnosed and cured individuals [172]. However, cost differences were driven primarily by the treatment price and not the cost of diagnosis. A recent systematic meta-analysis including 45 studies from both high-income countries and LMICs and comparing HCV POC viral load testing with laboratory-based RNA testing has informed new WHO recommendations to adopt POC HCV testing as an alternative to lab-based platforms in order to promote better linkage to care [173]. This meta-analysis found that on-site POC testing and mobile POC testing had treatment uptakes of 77% (72–83%) and 81% (60–97%), respectively, compared with 53% (31–75%) for standard laboratory testing.

### 3.4. Hepatitis D Virus

HDV, also known as hepatitis delta virus, is a satellite virus from the Kolmioviridae family that is dependent upon HBV for replication. The 1.7 kb circular RNA genome of HDV is the smallest of any known agent that infects humans. HDV is categorized into eight genotypes (1–8) [174]. It encodes a single gene that can be expressed in two forms, the small HDV antigen (HDAg) and the large HDAg. Virions are composed of these antigens and the viral genome that is packaged within lipid membranes containing HBsAg, expressed by HBV [175]. Like HBV, HDV is transmitted through contaminated blood products, and risk factors include unsafe sexual and injection practices and birth to a mother infected with HBV and HDV. There are an estimated 12 million people who have or have had an HDV infection globally [10]. The clinical course of HDV infections depends on when the infection occurs in relationship to HBV infection. Co-infections of HBV and HDV at the same time are often self-limiting, with less than 5% of cases becoming chronic, but they can cause fulminant hepatitis in some cases [7,176]. Superinfections by HDV in people with chronic HBV will lead to chronic HDV infections in 70–90% of cases [7,177]. These superinfections can exacerbate the signs and symptoms of hepatitis and lead to an earlier onset of cirrhosis. Vaccination against HBV also prevents HDV infection. Current interferon therapies for HDV are ineffective; however, bulevirtide and other HDV-specific therapies are being investigated [175,176].

Diagnostic markers for HDV infection include anti-HDV, HDAg, and HDV RNA, where the presence of HDV RNA or HDAg in the blood is used to diagnose current HDV infections. There are no FDA- or WHO-prequalified laboratory or point-of-care tests for either marker. Multiplex tests that detect both HDV and HBV markers would be ideal due to the intimate association between these two viral infections.

Of the five hepatitis viruses, HDV has received the least attention in relation to POC test development. An LFT for the detection of IgG anti-HDV antibodies from serum or plasma within 20 min was recently reported [178]. This test exhibited 94.6% (91.6–96.5%) sensitivity and 100% (97.4–100%) specificity when evaluated using a collection of 474 patient samples, including a range of anti-HDV titers and HDV genotypes. Additionally, the anti-HDV test was able to be multiplexed with lateral flow detection of HBsAg to allow for simultaneous diagnosis of HBV and identification of exposure to HDV. An RT-LAMP method for the detection of HDV RNA has also been developed and was able to confirm all HDV RNA-positive samples and identified more positive samples than enzyme immunoassays did for HDAg or anti-HDV [179]. For POC testing purposes, it would need to be integrated into simpler sampler processing and detection approaches.

### 3.5. Hepatitis E Virus

HEV is a non-enveloped, positive-sense RNA virus from the Hepeviridae family. It has a 7.2 kb genome and is genetically classified into at least eight genotypes (1–8), although only genotypes 1–4 infect humans [180,181]. The HEV genotypes are antigenically similar and comprise a single serotype. HEV is transmitted via the fecal–oral route and caused an estimated 19 million global infections in 2017 [11]. The primary source of infection and the epidemiological profile often differ by the development status of a country. In low- and middle-income countries, sewage contamination of drinking water can lead to large-scale outbreaks, typically involving HEV genotypes 1 and 2 [4]. In high-income countries, HEV infection is sporadic. In these places, improved sanitation and hygiene have made zoonotic transmission from the consumption of undercooked pork and game the main source of infections, typically with genotypes 3 and 4 [182]. HEV infections are often asymptomatic. When symptoms do occur, they appear one to two months after exposure and are generally self-limiting. Some hepatitis E cases can be severe, causing fulminant hepatitis and case–fatality rates between 1 and 3% [4]. HEV is particularly dangerous to pregnant women, where mortality rates of up to 30% have been reported [4,183]. Chronic HEV infections lasting longer than 6 months have occasionally been observed, but these are typically in immunocompromised patients infected with HEV genotype 3 [184]. A vaccine for HEV has been approved in China but is not available elsewhere [185]. There are no approved HEV therapeutics, although ribavirin has shown promise, and others are in development [186,187].

During HEV infection, both IgG and IgM anti-HEV antibodies can be detected about three to four weeks after exposure. The IgG antibodies are long-lived, while the IgM antibodies wane over an approximately 6-month period. Therefore, IgM anti-HEV is commonly used to identify acute infections. HEV is found in the blood and shed in stool of infected persons [188]. Both blood and stool can be used for diagnosing current HEV infection via HEV RNA or HEV antigen.

Commercial POC tests for the detection of IgM anti-HEV antibodies do exist; however, none have been approved by the FDA or prequalified by the WHO. Point-of-care testing for HEV would be particularly useful for rapidly identifying HEV outbreaks to allow for timely interventions that could minimize the scope of the outbreak. Additionally, POC tests could find use for screening water supplies and meat products from potentially infected animals.

The Assure HEV IgM rapid test (MP Biomedicals, Irvine, CA, USA) is an LFT that can detect IgM anti-HEV from serum, plasma, or whole blood in 15 min. Independent evaluations using serum or plasma samples have found sensitivities ranging from 92.6 to 96.7% and specificities ranging from 98.6 to 100% [187,188,189]. This test may have decreased sensitivity, 82% (68.6–91.4%), when used with samples from sporadic genotype 3 HEV infections and decreased specificity in samples containing high levels of rheumatoid factor [189,190]. There is often poor concordance among laboratory IgM anti-HEV tests, so reported sensitivities and specificities may need to be interpreted with caution [191]. Another test, the HEV IgM Rapid Test (Wantai Biopharm, Beijing, China), performs similarly to the Assure HEV IgM Rapid Test, with sensitivity above 90% [192]. This test was shown to have lower sensitivity, 73.3% (55.4–91.2%), when used with immunocompromised patients [193]. To evaluate the performance of these IgM anti-HEV LFTs as POC tests for the diagnosis of HEV, additional research should focus on the use of these tests with fingerstick blood specimens, rather than serum or plasma.

Researchers have developed tests for the detection of HEV capsid antigen or HEV RNA that have format attributes that are attractive for POC use. Unlike IgM anti-HEV, these markers would allow for the direct identification of HEV infection or environmental contamination. An LFT has been reported to detect HEV capsid antigen in 15 min with a sensitivity of 92% and a specificity of 100% [194]. This test uses a fluorescent tag that can be observed by eye or with a fluorimeter, rather than a colorimetric tag, for detection. It is compatible with both stool and serum samples and has a limit of detection equivalent to approximately 1000 HEV RNA genome copies per milliliter. For HEV RNA detection, RT-LAMP protocols have been reported [195]. One method using gold nanoparticles for colorimetric visual identification of DNA product amplification can be completed in less than 60 min and can detect samples with as little as 10 HEV RNA copies per reaction [196]. However, this method has not been evaluated with POC-compatible techniques for extracting HEV RNA or with whole blood or stool samples.

Isothermal nucleic acid amplification methods have also been adapted for use to detect HEV RNA from swine and shellfish samples. Pigs are a natural reservoir of HEV, and shellfish grown in contaminated waterways can concentrate viruses, such as HEV. Using nucleic acids extracted from pig bile or shellfish, RT-LAMP can detect approximately 10 HEV RNA copies per microliter of extract in 60 min using turbidity or colorimetric techniques for visual identification of DNA amplification [197,198]. An assay using RT-RPA with homogenized pork livers was found to have a limit of detection of 34 HEV RNA copies per microliter of extracted sample in only 20 min using either real-time fluorescence measurements or lateral flow test strips [199]. Each of these studies uses laboratory-based methods for sample homogenization and nucleic acid extraction due to the inherent complexity of the shellfish and liver sample types. While these isothermal methods are simpler and faster than conventional RT-PCR for detection of HEV RNA, simplifications to the sample preparation steps will be required before they are compatible with POC use to rapidly identity contaminated food items or animals.

Most large-scale HEV outbreaks occur due to contaminated sources of drinking water. However, rapid methods for HEV detection have not been applied to these sample types. Sensitivity may be an issue for this sample type due to the low levels of virus that are typically found in contaminated drinking water [200]. Laboratory testing of water sources for viruses often requires the lengthy concentration of liters of sample to smaller volumes that are compatible with extraction and amplification methods [201,202]. Simplifying these procedures for use at the POC will be difficult, although at least one method for performing virus concentration from water samples in the field with simple and inexpensive equipment has been described [203]. With technological advances, field testing of water sources may allow for drinking water screening or for the faster identification of outbreak sources.

## 4. Clinical Markers of Hepatitis

Symptoms of acute viral hepatitis, such as fever, nausea, and vomiting, can resemble those of other viral infections. Clinical signs of liver disease or dysfunction include high serum levels of bilirubin and/or liver enzymes like ALT. Identification of these clinical signs can indicate that testing for viral hepatitis infections is justified. While no commercial POC tests for elevated bilirubin or ALT levels exist, tests that are compatible with POC use have been developed. Bilirubin is an intermediate product formed during the catabolism of heme that can build up during liver disease. A bilirubin test that uses plasma separation cards and a battery-operated device for measuring absorbance can measure clinically relevant bilirubin levels from a few drops of whole blood in less than 5 min [204]. During liver disease, liver enzymes, such as ALT, are released into the bloodstream from damaged hepatocytes. A paper-based test that uses plasma separation and colorimetric semiquantitative measurement of ALT activity allows for assessment of whether ALT levels are elevated in serum or fingerstick blood samples in 30 min [205]. While use of this test with serum samples correlated well with a reference laboratory test, measurements made using whole blood samples were systematically lower. ALT levels above 40 IU/L can be used to evaluate whether HBV treatment should be initiated [80]. An LFT that uses ALT-specific antibodies has been characterized for its ability to identify plasma or whole blood samples with ALT levels above 40 IU/L [206]. The interpretation of results can be either quantitative using a handheld reader or semiquantitative using visual comparison to a standard. This method generally has a good predictive performance for determination of eligibility for HBV therapy compared with laboratory tests. The continued development of POC tests for measuring levels of bilirubin or ALT in blood samples is needed, because it may make diagnosis of liver disease and clinical decision making regarding viral hepatitis infections faster and able to be performed in resource-limited settings.

## 5. Conclusions

The five hepatitis viruses, HAV, HBV, HCV, HDV, and HEV, are major public health threats that cause millions of new cases and high levels of mortality each year. In general, diagnosis of these viruses is performed in laboratories, which leads to underdiagnosis due to a lack of access to testing or loss of patients to follow-up with testing results. Point-of-care testing methods for antibodies, antigens, and nucleic acids, which are rapid, inexpensive, and accurate, represent promising approaches for improving patient diagnosis, preventing the spread of disease, monitoring food and water for contamination with HAV or HEV, and improving linkage to care and treatment for chronic HBV and HCV infections. Rapid tests have been developed for many of the markers used in hepatitis virus diagnosis, with performance characteristics that are similar to their corresponding laboratory-based tests. Commercially available POC tests for HBsAg and anti-HCV are widely available and in use, while other markers such as viral hepatitis nucleic acids have only recently begun to be developed or made available. With additional improvements in cost, performance, and usability, the widespread adoption of POC tests could provide important tools for making progress towards the 2030 WHO viral hepatitis elimination goals.

Disclaimers: The findings and conclusions in this report are those of the authors and do not necessarily represent the official position of the Centers for Disease Control and Prevention. Use of trade names and commercial sources is for identification only and does not constitute endorsement by the U.S. Department of Health and Human Services or the U.S. Centers for Disease Control and Prevention.

## Figures and Tables

**Table 1:** Interpretation of hepatitis virus markers.

Virus	Marker	Interpretation
HAV	IgG anti-HAV	past HAV infection or HAV vaccination
	IgM anti-HAV	current or recent HAV infection
	HAV RNA	current HAV infection
	HAV capsid antigen	current HAV infection
HBV	IgM anti-HBc	acute HBV infection
	total anti-HBc	current or resolved HBV infection
	anti-HBs	resolved HBV infection or HBV vaccination
	anti-HBe	partial immune control of HBV infection
	HBV DNA	current HBV infection
	HBsAg	current HBV infection
	HBeAg	early-stage or chronic pre-seroconversion HBV infection
HCV	anti-HCV	current or resolved HCV infection
	HCV RNA	current HCV infection
	cAg	current HCV infection
HDV	anti-HDV	current or resolved HDV infection
	HDV RNA	current HDV infection
	HDAg	current HDV infection
HEV	IgG anti-HEV	past HEV infection or HEV vaccination
	IgM anti-HEV	current or recent HEV infection
	HEV RNA	current HEV infection
	HEV capsid antigen	current HEV infection

**Table 2:** WHO-prequalified point-of-care tests for hepatitis virus markers.

Test Analyte	Test Name (Company)	Sample Types	Time (min.)
HBsAg	Determine HBsAg 2 (Alere Medical, Waltham, MA, USA)	venous or capillary blood, plasma, or serum	15–30
HBsAg	Bioline HBsAg (Abbott Diagnostics, Abbott Park, Il, USA)	venous blood, plasma, or serum	20
anti-HCV	Rapid Anti-HCV Test (InTec Products, Xiamen, China)	venous or capillary blood, plasma, or serum	15–20
anti-HCV	Standard Q HCV Ab Test (SD Biosensor, Suwan, Republic of Korea)	venous or capillary blood, plasma, or serum	5–20
anti-HCV	OraQuick HCV Rapid Antibody Test (OraSure Technologies, Bethlehem, PA, USA)	venous or capillary blood, plasma, serum, or oral fluids	20–40
anti-HCV	Bioline HCV (Abbott Diagnostics, Abbott Park, IL, USA)	venous or capillary blood, plasma, or serum	5–20
HCV RNA	Genedrive HCV ID kit (Genedrive, Manchester, United Kingdom)	plasma and serum	90
HCV RNA	Xpert HCV Viral Load (Cepheid, Sunnyvale, CA, USA)	plasma or serum	105
HCV RNA	Xpert HCV VL Fingerstick (Cepheid, Sunnyvale, CA, USA)	venous or capillary blood	60–75
